# Fetal Distress and Neonatal Death After Thoracoamniotic Shunting Therapy Due to Hydrops Associated With Transient Abnormal Myelopoiesis

**DOI:** 10.7759/cureus.28991

**Published:** 2022-09-09

**Authors:** Junsuke Muraoka, Nozomi Yoshimoto, Ayako Ohsawa, Satoshi Matsuzawa, Shinji Katsuragi

**Affiliations:** 1 Obstetrics and Gynecology, University of Miyazaki, Miyazaki, JPN

**Keywords:** trisomy 21, transient abnormal myelopoiesis, thoracoamniotic shunting, hydrops fetalis, adverse event

## Abstract

We present the case of a pregnant woman who developed fetal bradycardia, which required an emergency cesarean delivery immediately after thoracoamniotic shunting (TAS), resulting in neonatal death four hours after birth due to hemodynamic deterioration. A 35-year-old Japanese female was referred to our hospital at 30^+0^ weeks of gestation due to fetal hydrops. Blood and ultrasonography examinations suggested that transient abnormal myelopoiesis (TAM) due to trisomy 21 could contribute to the development of hydrops fetalis. We performed thoracocentesis and TAS replacement as well as chromosomal testing of the amniotic fluid. However, the fetus developed persistent bradycardia soon after the treatment, and a sonographic examination revealed a recurrence of fluid retention in the thoracic cavity. A 1,558-g male neonate received life-saving resuscitation after being born via emergency surgery. Blood analysis revealed the occurrence of TAM in utero. At autopsy, there were no injuries to the intrathoracic organs. The G-banded karyotype revealed trisomy 21 (47, XY, +21). Our case offers new perspectives on providing prenatal information about potential complications to family members and selecting fetuses for TAS in the case of trisomy 21 complicated with TAM.

## Introduction

Thoracocentesis and thoracoamniotic shunting (TAS), in utero procedures for improving fetal hydrothorax or hydropic status, have been widely performed [1,2]. The aim of the treatment is to prevent or attenuate fetal lung hypoplasia and/or circulatory impairment due to hydrothorax or hydrops. The first Japanese trial of TAS for fetal pleural effusion using a double-basket catheter described the efficacy and safety of the procedure as a highly advanced medical treatment [3]. Subsequently, several studies reported that TAS contributed to a decrease in fetal mortality; the survival rates of fetuses with and without hydrops after TAS were reported to be approximately 70% and 100%, respectively [4,5].

Pregnant women with a singleton pregnancy between 18^+0^ and 33^+6^ weeks of gestation were eligible for TAS when indicated. Fetal karyotyping was usually offered but was not essential for performing the treatment. The most common adverse events associated with TAS are catheter displacement, premature rupture of membranes, and threatened preterm labor [6]. However, in a Japanese survey, no life-threatening adverse events that occurred soon after TAS were reported [6]. Herein, we present a case of fetal distress necessitating an emergency cesarean delivery immediately after TAS, resulting in fetal hemodynamic deterioration and neonatal death four hours after birth. The parents of the patient provided written informed consent for this report and accompanying images.

## Case presentation

A 35-year-old Japanese para 2, gravida 1 female, who had an uneventful course until 28 weeks of gestation, was referred to our tertiary hospital at 30^+0^ weeks of gestation due to fetal hydrothorax. On the day of referral, fetal ultrasonography revealed fetal pleural effusion, ascites, anasarca, and hepatomegaly (enlarged ≥95 percentile). The fetal cardiac structure and contractility were normal. No other fetal anatomical abnormalities were detected. The peak systolic velocity of the middle cerebral artery was 65 cm/second (≥1.5 multiples of median), indicating fetal anemia. The flow of umbilical blood and the ductus venosus was normal. Maternal blood antibody screening was negative, and laboratory tests revealed no evidence of intrauterine infection, such as B19 parvovirus, coxsackievirus A or B, herpes simplex, cytomegalovirus, or toxoplasmosis. Fetal heart rate (FHR) monitoring revealed intermittent late deceleration with moderate variability and no acceleration. We diagnosed hydrops fetalis and proposed transient abnormal myelopoiesis (TAM) due to trisomy 21 as a possible cause. After receiving informed consent from the patients, we performed thoracocentesis and TAS replacement as well as chromosomal testing of the amniotic fluid.

**Figure 1:**
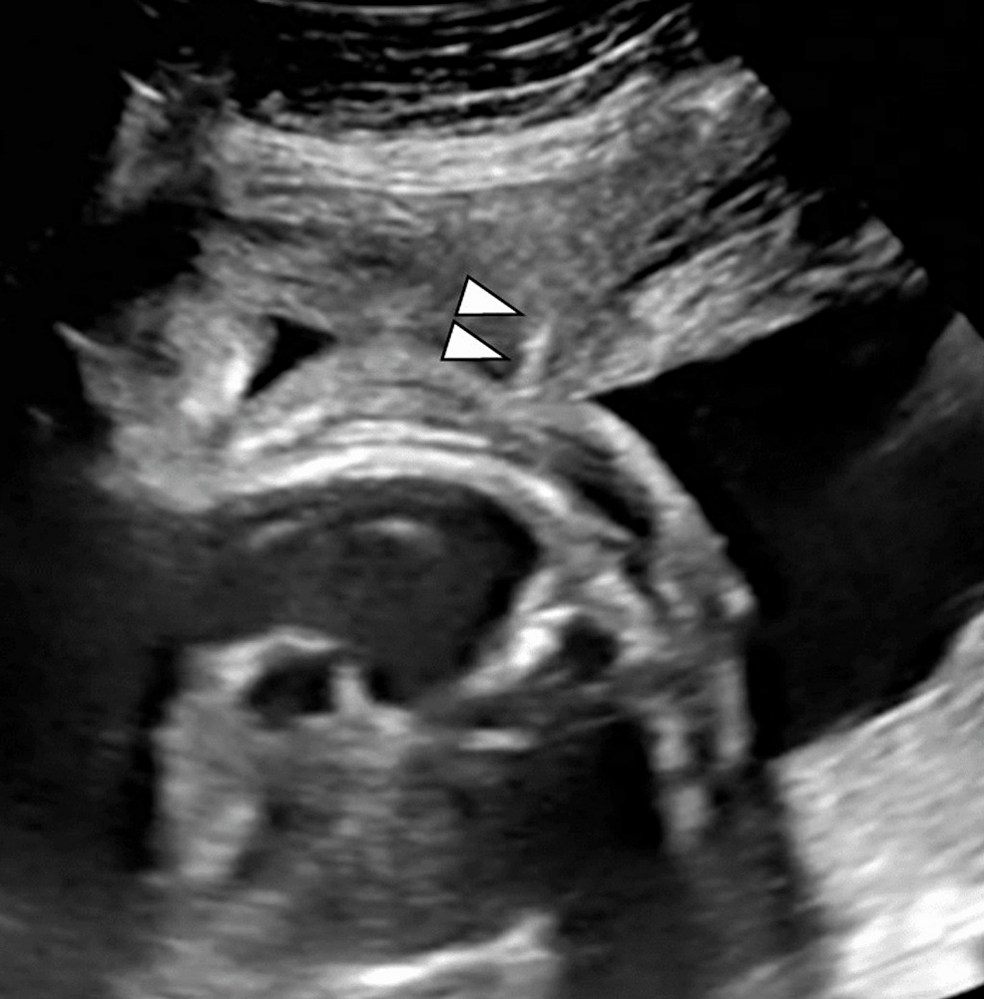
Transabdominal ultrasound image of catheter puncture during TAS. Arrowheads: tip of the shunting catheter TAS: thoracoamniotic shunting

**Figure 2:**
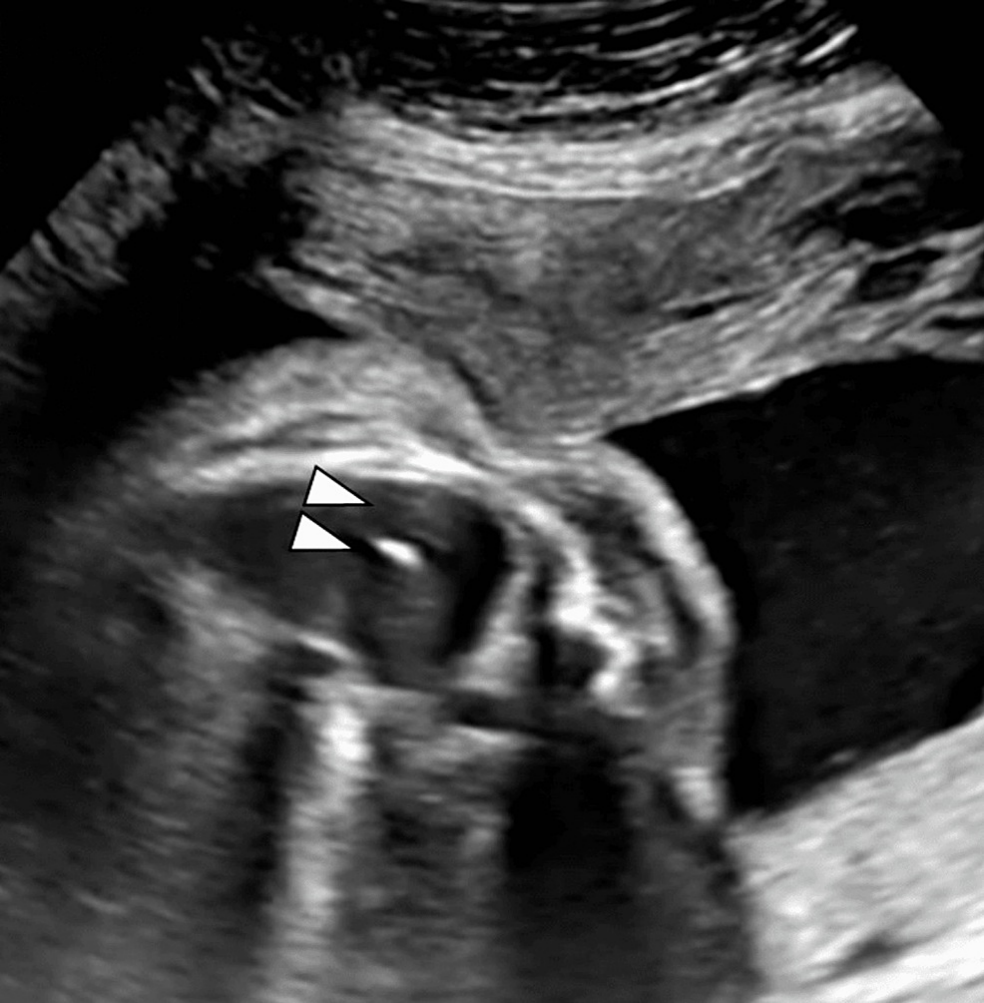
The tip of the catheter was transmitted to the pleural space. Arrowheads: tip of the shunting catheter

General and epidural anesthesia were applied to alleviate maternal pain. A 16-gauge puncture needle with a trocar (5 Fr) (outer diameter: 1.6 mm) was inserted from the right posterolateral thoracic wall of the fetus under ultrasound guidance (Figures 1, 2).

**Figure 3:**
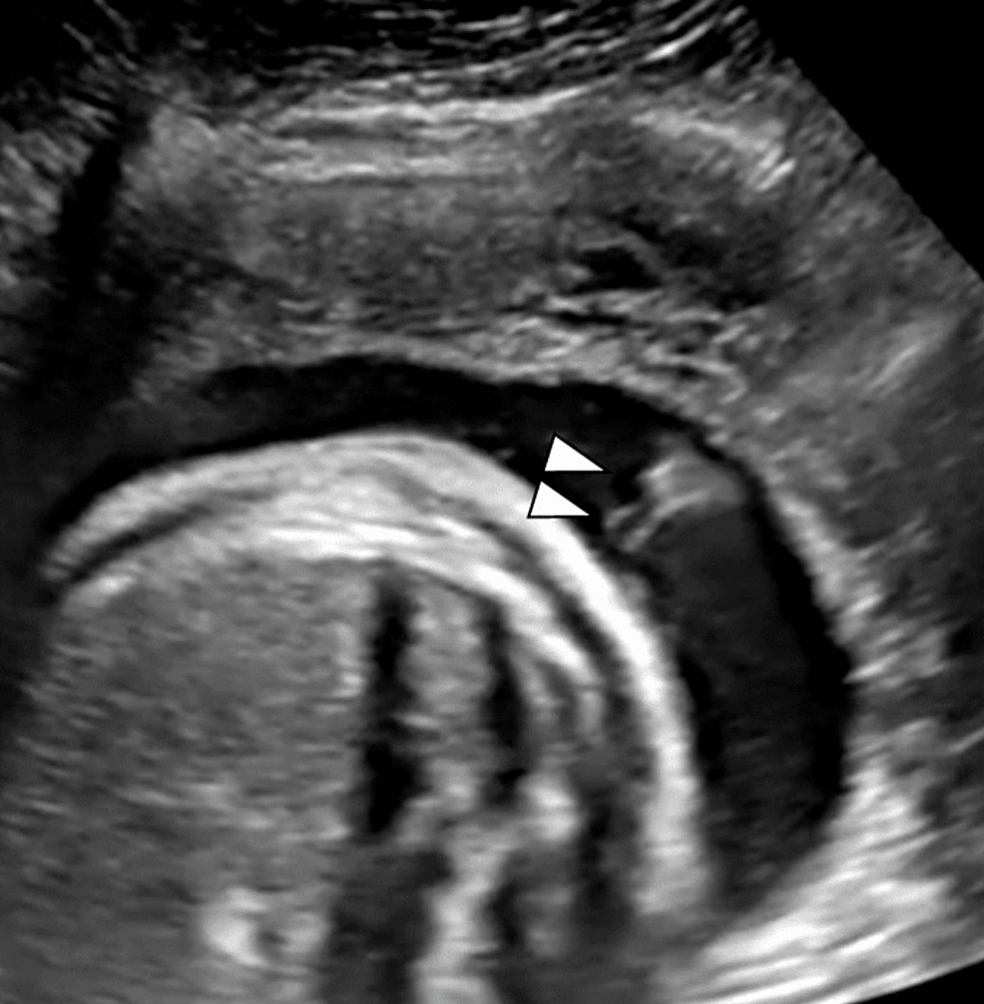
The shunting catheter was placed in the fetal chest cavity. Arrowheads: rear end of the shunting catheter in the amniotic fluid cavity

**Figure 4:**
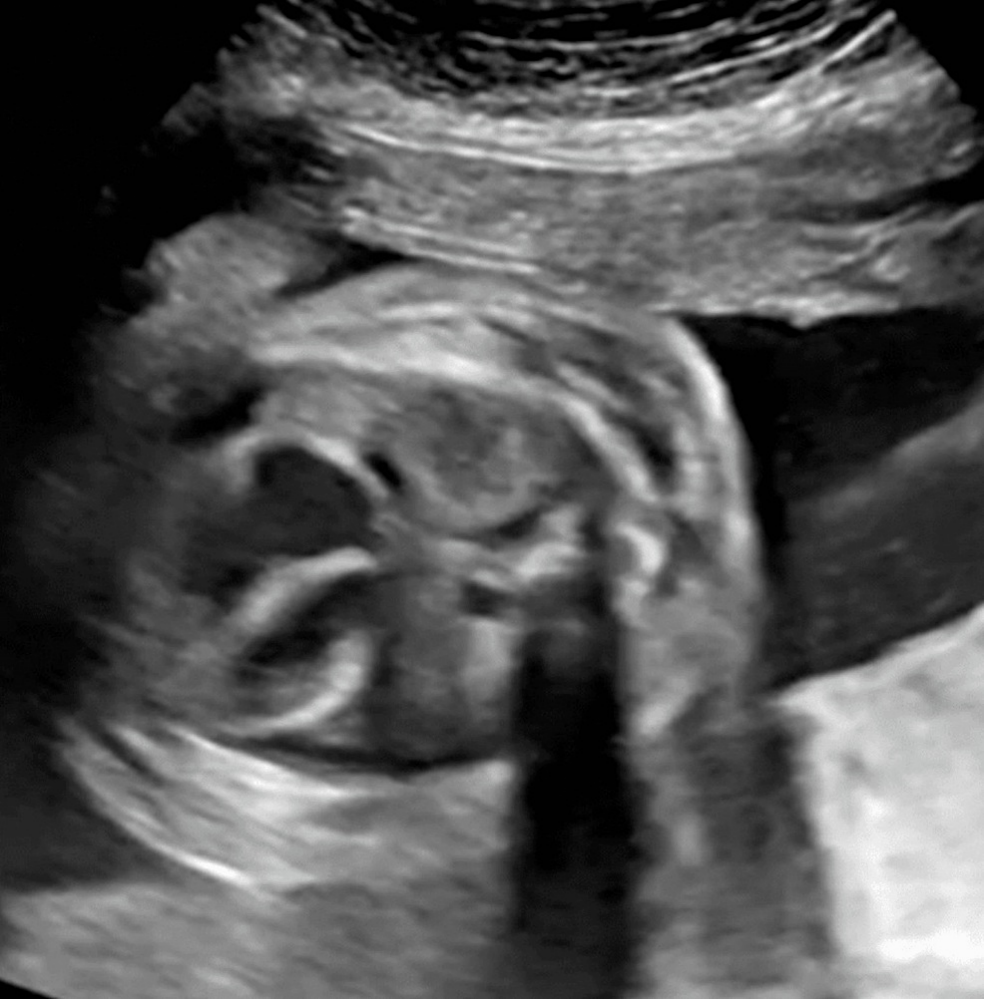
The right-side lung of the fetus was expanded after TAS. TAS: thoracoamniotic shunting

After 8 mL of yellow-colored serous effusion was removed, a double-basket catheter (Hakko Co., Nagano, Japan) was placed with a one-time aspiration throughout the procedure. The effusion contained 92% of lymphocytes. Almost all pleural effusions were drained from the thoracic cavity in 10 minutes, and the right-side lung was well expanded (Figures 3, 4).

Regular 150 beats/minutes of FHR were confirmed when the procedure was completed. However, the fetus developed persistent bradycardia of <80 beats/minute several minutes after the treatment, and a sonographic examination revealed a recurrence of fluid retention in the right thoracic cavity. We performed an emergency cesarean section due to non-reassuring fetal status. During delivery, the shunt tube detached from the infant’s body.

A 1,558-g male neonate was born and received life-saving resuscitation, including intubation, adrenaline administration, and right-side chest drainage. Neonatal blood gas analysis revealed a pH level of 6.71 and a hemoglobin level of 2.8 g/dL. Fibrinogen levels and antithrombin activity were lower than sensitivity, indicating coagulopathy. Although rapid transfusion of red blood cells was repeated, bloody discharge flowed outwardly through the chest drainage. Neonatal peripheral blood analysis revealed a differential leukocyte count with 68% blasts, implying the occurrence of TAM in utero. Approximately four hours after birth, the infant died.

**Figure 5:**
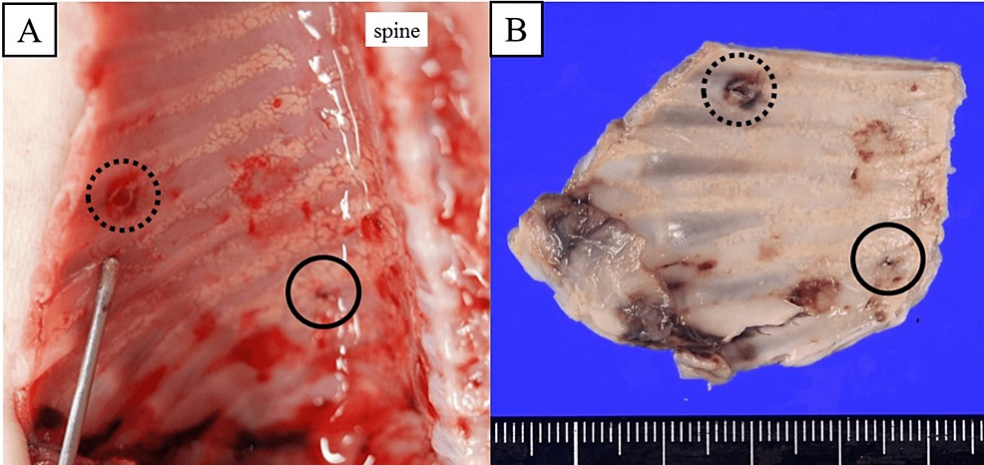
Pathologic findings in the right thorax at autopsy. The puncture scars were viewed inside the right thorax at autopsy before (A) and after (B) formalin fixation. Solid circles: TAS scars; dotted circles: postnatal chest drainage scars TAS: thoracoamniotic shunting

The G-banded karyotype revealed trisomy 21 (47, XY, +21). At the autopsy, two puncture points were noted on the right side of the chest wall (Figure 5). One was located adjacent to the thorax, and the other was located posterior to the thorax, which seemed to have been caused by chest drainage and TAS, respectively. There were no traumatic injuries to intrathoracic organs, including the heart, lungs, or mediastinum.

The committee board of the Division of Medical Safety Management at the University of Miyazaki Hospital discussed establishing future preventive measures.

## Discussion

This case exhibited non-reassuring fetal status requiring emergency cesarean delivery immediately after TAS, resulting in neonatal death. Neonatal hemodynamic deterioration with coagulopathy indicated exacerbated TAM in utero. There had been no reports of severe procedure-related complications of TAS in the Japanese survey; therefore, a detailed investigation of our case seemed critical. Moreover, our case offers new perspectives on providing prenatal information about potential complications to family members in the case of trisomy 21, complicated with TAM and having a risk of selecting the fetuses as participants in TAS.

It is debatable whether TAS should be performed as the initial treatment for fetal pleural effusions or hydrops because TAS is originally conducted after thoracocentesis. The Japanese Society of Fetal Therapy previously conducted a multicenter prospective study for TAS using a double-basket catheter [3]. The study included fetuses whose chylothorax showed retention to pre-procedure levels within seven days after the initial thoracocentesis. Shunts have recently been used as the initial intervention [7]. Given that fetal hypoxic status expressed in FHR patterns seemed urgent, we conducted TAS in addition to thoracocentesis through the same access as a favorable intervention instead of repetitive procedures at intervals. We also expected to see an improvement in circulatory impairment due to hydrops from TAS.

Associated aneuploidy has been reported in 7%-12% of hydrops fetalis and worsens the outcomes [4]. Although thoracocentesis or TAS is performed on hydropic fetuses associated with lethal chromosomal abnormalities, such as trisomy 13 or 18, a favorable prognosis cannot be predicted. Meanwhile, some previous studies included infants with trisomy 21 as participants of TAS. Yinon et al. demonstrated that two hydropic fetuses with trisomy 21 showed a resolution of hydrops after TAS but died of persistent pulmonary hypertension during neonatal periods [8]. In contrast, the study by Takahashi et al., which provided a basic Japanese protocol for TAS, included no fetuses with trisomy 21 as participating patients [3]. Thus, it would be an acceptable choice to perform TAS for fetuses suspected of complicating trisomy 21.

TAM, a rare and unique disorder affecting Down syndrome, is a fetal hematopoiesis disease that can resolve spontaneously during fetal and neonatal lives [9,10]. TAM has variable manifestations in utero ranging from intrauterine fetal death or hydrops fetalis to clinically silent disease. Prognostic factors associated with early death include hyperleukocytosis, hepatomegaly, abnormal liver function tests, prematurity, ascites, coagulopathy, and failure to normalize blood counts [10]. The patient herein manifested hepatomegaly and anemia in utero, followed by abnormal blood counts and coagulopathy after birth, which may have resulted in neonatal death due to exacerbated TAM. Although technically difficult, cordocentesis may be a viable option for confirming fetal anemia or examining blast counts.

Double-basket catheters are the only approved equipment for TAS in Japan. The catheters are expected to be less invasive for the fetus because they are soft and thin. At present, Japanese studies have found no lethal adverse events associated with TAS for fetuses or mothers [6]. Our case first exhibited an extremely severe adverse event that occurred shortly after TAS. An autopsy revealed no injuries to vessels or organs by TAS procedure; therefore, TAS would be sufficiently influential to exacerbate the potential hematological malfunction of TAM.

## Conclusions

We present the first case of fetal distress and neonatal death immediately after TAS. TAS therapy can lead to lethal complications for fetuses and neonates. Sufficient discussion about the indication for TAS should be made in assuming hydrops related to TAM in Down syndrome. The fetus with hydrops should be inspected carefully for evidence of TAM, and physicians should provide information to family members about the lethal complications of the procedure and the prognosis expected.
